# The high-affinity human IgG receptor Fc gamma receptor I (FcγRI) is not associated with vascular leakage of dengue

**DOI:** 10.1186/s12952-014-0020-6

**Published:** 2015-01-08

**Authors:** Zaiharina Mohamad Zamberi, Zuraihan Zakaria, Abu Thalhah Abdul Aziz, Benedict Sim Lim Heng, Masliza Zaid, Christopher Lee Kwok Chong, Fadzilah Mohd Noor, Sazaly Abu Bakar, Hoh Boon Peng

**Affiliations:** Institute of Molecular Medical Biotechnology (IMMB), Faculty of Medicine, Universiti Teknologi MARA, Sungai Buloh Campus, 47000 Sungai Buloh, Selangor Malaysia; Hospital Sungai Buloh, Jalan Hospital, 47000 Sungai Buloh, Selangor Darul Ehsan Malaysia; Microbiology Unit, Centre for Pathology Diagnostic and Research Laboratories (CPDRL), Level 1, Clinical Training Centre (CTC), Faculty of Medicine, Universiti Teknologi MARA, Sungai Buloh Campus, Jalan Hospital, 47000 Sungai Buloh, Selangor Malaysia; Drug and Discovery Research Core, Universiti Teknologi MARA, Shah Alam, 40450 Shah Alam, Selangor Malaysia; Department of Medical Microbiology, Faculty of Medicine, University of Malaya, 50603 Kuala Lumpur, Malaysia; Tropical Infectious Disease Research and Education Centre, Faculty of Medicine, University of Malaya, 50603 Kuala Lumpur, Malaysia

**Keywords:** Dengue, Vascular leakage, FcγRI, ELISA

## Abstract

**Background:**

Dengue is a major public health problem in many tropical and sub-tropical countries. Vascular leakage and shock are identified as the major causes of deaths in patients with severe dengue. Studies have suggested the potential role of Fc gamma receptors I (FcγRI) in the pathogenesis of dengue. We hypothesized that the circulating level of Fcγ receptor I could potentially be used as an indicator in assisting early diagnosis of severe dengue.

**Results:**

A selected cohort of 66 dengue patients including 42 dengue with signs of vascular leakage, and 24 dengue without signs of vascular leakage were identified and were afterwards referred to as ‘cases’ and ‘controls’ respectively. Thirty seven normal healthy controls were also recruited in this study. The circulating level of FcγRI was quantified from the serum using enzyme-link immunosorbent assay (ELISA). The levels of FcγRI in both groups of patients with and without vascular leakage were found to be significantly higher than the normal healthy controls (P < 0.001). However, there was no significant difference found between patients with vascular leakage and those without vascular leakage (p = 0.777).

**Conclusion:**

We suggest that FcγRI is not associated with the vascular leakage in dengue. However, further studies are necessary to delineate the role of FcγRI in antibody-dependent enhancement (ADE) mechanism.

## Background

Dengue is caused by infection with one of the fours related but antigenically distinct serotypes namely, DENV-1, DENV-2, DENV-3, and DENV-4. Globally, dengue infections have risen from less than 1,000 cases in 1955 to almost 1,000,000 cases in 2007. Approximately 2.5 billion infected individuals live in endemic areas, of which 50 million individuals are infected annually [1]. Dengue cases in Malaysia have risen dramatically, with approximately 6,500 cases in 1995, to more than 49,000 cases in 2007 [2]. Dengue virus (DENV) infection causes a spectrum of clinical manifestations, ranging from asymptomatic, undifferentiated fever, dengue fever (DF) to a more severe form of syndrome - dengue haemorrhagic fever (DHF), characterized by plasma leakage and haemorrhage, representing a life-threatening complication [3,4].

The severe form of dengue contributes approximately 5 - 10% of all reported cases, and could be lethal if it is not treated properly [5,6]. Secondary infection is identified as a predominant risk factor leading to the severe manifestation [7-9]. This can be explained partly by the hypothesis called “antibody-dependent enhancement” (ADE) mechanism in the host, which occurs when the neutralizing antibody generated during the first immune response binds to the DENV with different serotype during secondary infection but not being able to neutralize it. The DENV then unites with the heterotypic antibody, forming the virus-immunoglobulin complex and enters the host cells via Fc receptors. Subsequently, viral replication takes place [10]. This process triggers the “cytokine storm” cascade, which eventually directs the alteration of complement pathway, leading to the activation of inflammatory signalling cascade and further resulting in vascular leakage [11,12].

The Fc receptor is the principle component in ADE. It is an important immunoglobulin receptor that involves in regulating normal and pathological immunity [13]. The IgG receptors (FcγR) is the largest family of Fc receptor, encoding two low-affinity receptor groups, FCGR2 and FCGR3; and a unique high-affinity receptor, FCGR1 [14]. It was believed that FCGR1 mediated the phagocytosis that is involved in the removal of antibody-opsonized DENV [15].

Early detection of shock and other complications may reduce the morbidity and mortality of severe dengue [1]. Although intensive efforts have been made to study the earlier clinical pathophysiology of dengue by identifying the potential cause of DHF, the appropriate strategy for early detection and prediction on which patients would progress to dengue hemorrhagic fever (DHF) has not been established yet [16]. Given the potential role of FcγRI in ADE, we hypothesized that it could possibly be used as an indicator in assisting the early diagnosis of dengue patients developing vascular leakage. Therefore in this study, we attempted to evaluate circulating FcγRI levels of dengue patients with vascular leakage versus without vascular leakage, presumably that the circulating FcγRI shaded from the cell surface in the course of infection.

## Results and discussion

Of the 95 dengue patients recruited, 29 were excluded from further analysis due to: (i) the negative results in both IgM and IgG serology tests; and (ii) the presence of co-infection. Sixty-six (66) dengue subjects (41 males and 25 females, of which 53 were Malays, 5 Chinese and 8 Indians), and 37 normal subjects (11 males and 26 females, of which 28 were Malays, 3 Chinese and 6 Indians) remained for the analysis (Table 1). All subjects were presented with fever greater than 37°C, accompanied by two or more of the following manifestations: abdominal pain, headache, vomiting, hepatomegaly, leukopenia or thrombocytopenia. Dengue causes a wide range of clinical manifestations. Therefore, although the subjects were clinically classified as uncomplicated DF, DF(WS), and SD, the focus was made on a single trait known to be the hallmark symptom of dengue hemorrhagic fever - vascular leakage, with the notion that the severe form of dengue can be prevented by preventing the vascular leakage of the patients.

**Table 1:** **Demographic data with average duration of illness and laboratory parameters of normal healthy subjects, controls and cases**

**Demographics**					**p value
		**Normal (healthy)**	**Control**	**Case**	
**Number of patients**		37	24	42	NA
**Gender**	**Female**	26	7	18	0.003
	**Male**	11	17	24	
**Age (years)**	**11 – 20**	1	4	11	0.677
	**21 – 30**	27	12	15	
	**31 – 40**	7	3	9	
	**41 – 50**	1	3	6	
	**51 – 60**	1	1	1	
**Average duration of illness (DOI) (days)**		NA	5 (3 – 7)	5 (1 – 7)	0.856
**Body temperature, mean (°C)**		NA	38.5 (37.0 – 40.0)	38.1 (37.0 – 40.6)	NA
**Max Hct, mean**	**Female**	NA	38.0 (35.7 – 39.9)	42.9 (26.5 – 50.0)	<0.001
	**Male**	NA	42.7 (33.8 – 45.7)	48.7 (40.0 – 55.5)	

*NA, not applicable; **The indicated P value in the gender was calculated for both males and females in normal, control and case groups, indicating a nonrandom gender distribution in the groups; age refers to the distribution of normal, control and case groups (P<0.05).

The samples were categorized as “cases” when they presented any signs of vascular leakage; and “control” for those without signs of vascular leakage. Dengue with signs of vascular leakage can be defined when either patient has (i) a significant increased level of hematocrit (H_CT_) (20% above the baseline measurement, or >46% as indicated by the Ministry of Health Malaysia [1,17]; or, (ii) the presence of pleural effusion, and/or ascites. In clinical settings, H_CT_ level has been commonly used as an indicator for vascular leakage [12]. Based on our evaluation of the sample classifications, apparently 92.4% of the cases/controls classifications were in accordance with the clinical diagnosis, except for five subjects. All the five subjects were clinically diagnosed as DF, yet, there was a marked increment of H_CT._ Therefore, it was categorized as “cases”.

The maximum H_CT_ level between the cases and controls were significantly different (P < 0.001), supporting a well sample classification between these groups (Table 1). No significant difference was observed between the dengue groups by duration of illness (p = 0.856), thus we eliminated this as a potential confounding factor for the disease severity in this study.

**Figure 1:**
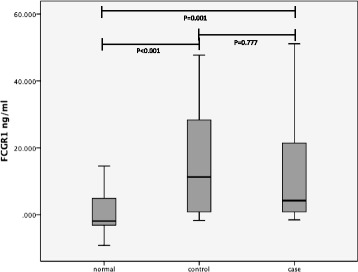
**Serum level of FcγRI measured by ELISA for normal healthy controls, controls and cases.** Asterisks indicate p value for normal healthy controls, controls and cases. P value is statistically significant (p < 0.001) in the control group compared to normal healthy controls. No statistically significant difference between cases and controls (P = 0.777).

The FcγRI levels were compared between the cases and controls, and then with the normal healthy controls (denoted as NC) (Figure 1). The FcγRI protein level was significantly higher (p < 0.001) in the control group compared to NC. It was also observed that the level of FcγRI in cases group was significantly higher (p = 0.001) than NC. However, there was no significant difference between the cases and controls (p = 0.777) (Figure 1).

The mortality of dengue patients could have been improved by early detection of dengue infection followed by intensive treatments of the patient [18]. However, one of the major challenges to the clinicians is to accurately identify patients who potentially develop vascular leakage, a hallmark symptom prior to the development of a severe form of dengue. Therefore, identification of biomarkers for early detection of vascular leakage in dengue is crucial for effective management of the dengue patients. To date, although several diagnostic methods are available for dengue diagnosis [19-22], none could accurately predict the propensity for dengue-infected patients to progress into vascular leakage.

The immunoglobulin FcRs play a critical role in the immune defense system by binding the Fc portions of antibodies and providing a link between humoral and cellular immune response [23]. FcγRI is an activating receptor that recruits the gamma subunit with immune-receptor tyrosine-based activating motif to phosphorylate kinases that signals for phagocytosis [24], pro-inflammatory responses [13], protection from bacteria [25], viruses [26] and helps in the removal of antibody-opsonized DENV [15]. Over the past decade, research in the field of Fc-receptors biology and their role during various phases of an immune response has been well established [27-29]. But for the first time, we presented that there was significant elevation of circulating FcγRI, presumably shaded from the cell surface in course of the disease, observed in patients with dengue. Our results demonstrated that FcγRI was increased in dengue patient, providing further supporting evidences of its involvement in the events of immune responses during dengue viral infection [15].

**Figure 2:**
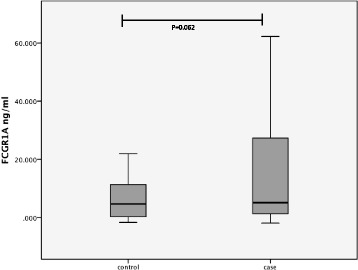
**Comparison of the circulating FcγRI level of the controls and cases using ELISA, representing the repeat analysis that involves samples collected within 4th – 7th day of duration of illness.** No statistical significance was observed between the two categories (P = 0.062).

In this study we also observed that the dengue patients who showed signs of vascular leakage; although not significant, on average, demonstrated a relatively lower FcγRI (Figure 1). Animal model study suggested that FcγRI deficient may lead to impaired phagocytosis of the immune complex function, hence resulting into the elevation of antibody responses [13]. This leads us to postulate that the reduced of FcγRI level in patients with vascular leakage causes elevation of antibody level, therefore increases the number of antibody-forming cells, which in turn increases the bridging of infections of DENV particles and FcR on cell surfaces. Consequently, it also increases the number of the infected cell. The reason why it is not significant between ‘cases’ and ‘controls’ is unclear though, and warrants further investigation in the future. We realized that the day of which the samples were collected from the day of onset illness (DOI), a potential confounding factors to this finding. Hence, we repeated the analysis by including the samples that was only collected within 4^th^ – 7^th^ duration of illness, and found no significant difference of FcγRI level (Figure 2).

The non-significance between the cases and the controls in this study could be associated to two downsides of the study, namely; (i) the sample size recruited; and (ii) the emphasis given only to subjects with vascular leakage may possibly lead to false negative finding.

## Conclusion

Our study shows a significant difference of FcγRI levels between dengue patients and normal healthy controls, but not between dengue patients with or without vascular leakage. Further understanding on the role of FcγRI in the mechanism of ADE upon FcγR mediated phagocytosis is crucial for the understanding of immunity and pathogenesis of vascular leakage, to further assisting in refining the vaccine development [30] and also the development of the biomarker for vascular leakage of dengue.

## Methods

### Ethics statement and study population

This study was reviewed and approved in accordance with the Ethical Committee of Universiti Teknologi MARA and Ministry of Health Malaysia, for the study of host genetics of dengue (Ministry of Health: NMRR-09-1128-4211; Universiti Teknologi MARA: 600-RMI (5/1/6)). Ninety-five (95) adult patients clinically diagnosed with dengue were recruited in this study. Diagnosis of dengue were confirmed by performing commercially available Dengue specific capture Enzyme-Linked Immunosorbent Assay kit (ELISA) IgG and IgM tests (PanBio, Australia) [31,32] to all 95 collected serum samples. Informed and written consent was obtained from the patients. Three millilitres (3 ml) of patients’ peripheral blood were collected. Patients who were diagnosed to be co-infected with other pathogens, or were negative for both IgG and IgM, were excluded from the study. All patients were admitted to the medical ward in Hospital Sungai Buloh during the year of 2010 to 2012. Subjects were clinically diagnosed as dengue fever (DF), dengue fever with warning sign [DF(WS)], or severe dengue (SD) by the clinicians from the hospital, following 2009 World Health Organization (WHO) case definition [1]. Demographic characteristics (i.e. age, gender and race) and clinical manifestations (i.e. day of fever, body temperature, bleeding manifestation, pleural effusion, ascites and abdominal pain) were gathered with routine hematological and biochemical laboratory test findings (i.e. full blood count, liver function test).

Additional blood samples were collected from 37 healthy volunteers to determine serum FcγRI antibody levels in the population, and these samples serve as normal healthy control subjects. Healthy volunteers were characterized as have not been infected with dengue that is the absence of any dengue antibody (IgM and/or IgG). Healthy volunteers were matched to patients according to age, gender and race that were selected from the community as a normal group.

### Quantification of FcγRI

Quantification of FcγRI level was carried out in duplicates on the serum samples using FcγRI pre-coated ELISA plates (i-DNA Technologies, Singapore). Standards and 100 μl of diluted serum sample (1:100 dilution) were added to their respective wells. The microtiter plate was pre-coated with antibody specific to FcγRI and the colour change was measured by using spectrophotometer. The concentration of FcγRI in the samples was then determined by comparing the optical density (O.D.) of the samples to the standard curve.

### Statistical analysis

Comparisons of the mean values within groups were carried out using one-way ANOVA, followed by Sidak post-test to identify the differences. Data analysis was carried out using SPSS for Windows version 20.0 (SPSS Inc. Chicago, IL, USA). P value of less than 0.05 was considered to be significant for all tests performed.
